# Acupuncture reduces neuroinflammation and apoptosis, regulates peripheral immunity, and modulates T-cell subset distribution in vascular dementia rats

**DOI:** 10.1186/s13020-025-01243-6

**Published:** 2025-11-26

**Authors:** Xinliang Wang, Xiaoxi Liu, Wenyu Zhang, Sai Qi, Jinyan Li, Ruiyu Li, Jingxin Guo, Yifan Zhang, Xuyang Feng, Xuezhu Zhang

**Affiliations:** 1 First Teaching Hospital of Tianjin University of Traditional Chinese Medicine, Tianjin, 300380 China; 2 National Clinical Research Center for Chinese Medicine Acupuncture and Moxibustion, Tianjin, 300380 China; 3 Tianjin University of Traditional Chinese Medicine, Tianjin, 301617 China; 4 Nursing College, Pingdingshan University, Pingdingshan, 467000 China; 5 Beijing University of Chinese Medicine Shenzhen Hospital (Longgang), Shenzhen, 518172 China

**Keywords:** Vascular dementia, Acupuncture, Neuroinflammation, Apoptosis, Peripheral immune

## Abstract

**Background:**

Vascular dementia (VD), primarily caused by cerebral hypoperfusion, is a major dementia subtype. Our previous studies demonstrated that acupuncture improves clinical outcomes in VD patients and modulates their peripheral immune responses. Nevertheless, the mechanistic interplay between acupuncture-mediated peripheral immunomodulation and cognitive enhancement remains to be elucidated.

**Methods:**

The cognitive abilities of rats were assessed using the Morris water maze (MWM), novel place recognition (NPR), and novel object recognition (NOR) tests. Neuronal injury and apoptosis in the hippocampal CA1 and CA3 regions were evaluated by hematoxylin and eosin (HE) staining and TUNEL assay. Immunofluorescence staining was performed to detect microglial activation markers (Iba-1 and CD68). Cytokine levels—including interleukin-1β (IL-1β), IL-6, tumor necrosis factor-α (TNF-α), IL-2, IL-17A, IL-4, IL-10, transforming growth factor-β (TGF-β), and IL-35—in hippocampal tissues and peripheral blood were quantified by enzyme-linked immunosorbent assay (ELISA). Western blotting was employed to analyze the expression of cleaved-caspase 3, caspase-3, Bcl-2, and Bax in rat hippocampal tissues. Flow cytometry was used to analyze the proportion, proliferation, and apoptosis of CD3⁺ T cells, CD4⁺ T cells, and CD8⁺ T cells in peripheral blood.

**Results:**

Acupuncture ameliorated cognitive impairment in VD rats, reduced hippocampal neuronal damage and apoptosis, downregulated pro-apoptotic proteins (cleaved-caspase 3 and Bax), and upregulated anti-apoptotic Bcl-2. Furthermore, it suppressed microglial activation markers (Iba-1 and CD68), decreased pro-inflammatory cytokines (IL-1β, IL-6, TNF-α, IL-2, IL-17A), and elevated anti-inflammatory cytokines (IL-4, IL-10, TGF-β, IL-35) in the brain. Simultaneously, acupuncture modulated peripheral inflammatory cytokine profiles, increased CD3⁺ T cell and CD4⁺ T cell proportions, and reduced T-cell apoptosis in peripheral blood of VD rats.

**Conclusions:**

Acupuncture improved cognitive impairment in VD rats and suppressed neuroinflammation and neuronal apoptosis; these benefits may be mediated, at least partially, through modulation of peripheral immunity.

**Supplementary Information:**

The online version contains supplementary material available at 10.1186/s13020-025-01243-6.

## Introduction

Vascular dementia (VD) refers to a group of acquired cognitive dysfunction syndromes characterized by cognitive and memory impairment, caused by a series of cerebrovascular diseases that damage brain tissue [1]. Globally, VD accounts for 20–30% of dementia cases among the elderly, ranking as the second most prevalent dementia subtype after Alzheimer’s disease (AD) [2]. It is expected that the number of VD patients worldwide will increase to 152 million by 2050, becoming one of the major public health challenges [3]. VD represents the only potentially reversible dementia under current clinical management [4], yet effective therapies remain limited. Consequently, elucidating its pathogenesis and developing novel treatments are critically imperative.

Previous studies have shown that neuroinflammation is indispensable in the occurrence and development of VD [5]. As the brain's resident immune cells, microglia maintain central immune homeostasis [6]. When exposed to pathological stimuli, microglia are promptly activated and polarized, releasing pro-inflammatory cytokines [7]. For instance, M1-phenotype microglia release interleukin-1β (IL-1β), tumor necrosis factor-alpha (TNF-α), and IL-6. These cytokines damage the blood–brain barrier (BBB), enhance the infiltration of inflammatory cytokines into the brain, and eventually cause neuronal injury and apoptosis [8, 9]. The hippocampus, a crucial area in the brain accountable for cognition, learning, and memory, is particularly susceptible to ischemic injury [10]. Chronic cerebral hypoperfusion (CCH) sustains microglial activation, exacerbating hippocampal neuronal dysfunction and accelerating cognitive decline in VD [11]. Meanwhile, the neuroinflammation induced by CCH can directly or indirectly activate the apoptotic pathway, thereby triggering neuronal apoptosis [12].

Studies have found that VD patients not only have neuroinflammation, but also peripheral inflammation and peripheral immune function abnormalities [13]. Moreover, the peripheral immune system has a profound influence on the central immune activity [14]. Peripheral inflammation can exacerbate neuroinflammation and neurodegenerative diseases. Intraperitoneal injection of high-dose lipopolysaccharide (LPS) induces systemic inflammation, causing behavioral alterations and cognitive impairment in mice [15], which coincides with microglial activation, elevated pro-inflammatory cytokines (TNF-α, IL-1β), and reduced anti-inflammatory cytokines (IL-4, IL-10) in the brain [16]. Elevated plasma IL-1β in sepsis-related encephalopathy patients exacerbates memory deficits [13, 17]. T cells, as the main effector cells of the human immune system, play an important role in regulating the immune balance of the body. Studies have demonstrated that T cells play a crucial role in maintaining normal cognitive function. For instance, peripheral T-cell deficiency can impact cognitive function in rats, and cognitive function is restored after T-cell transplantation [18]. The inhibition of CD4^+^ T-cells can lead to hippocampal neuron damage and cognitive impairment [19]. These data suggest that correcting peripheral inflammation and maintaining immune homeostasis are crucial for mitigating neuroinflammation and improving cognitive function. This provides a new idea for the prevention and treatment of VD.

Acupuncture, a traditional Chinese medicine therapy, ameliorates neurological diseases including VD [20, 21]. The functional activity of Qi within the “Sanjiao” declines with age, resulting in functional impairments across multiple tissues and organs and ultimately contributing to the development of dementia [22]. As the pathway responsible for the ascending, descending, exiting, and entering of Qi, blood, essence, and body fluid, the “Sanjiao” also serves as the source of their production. Additionally, as the commander of Qi activity, the “Sanjiao” governs the functional activities of five zang and six fu-organs. Thus, the abnormal function of “Qi activity in Sanjiao” is the basic mechanism for aging [22]. Based on the functional characteristics of Qi in the “Sanjiao” and extensive clinical experience, we have selected the following acupuncture points for preventing and treating dementia and delaying brain aging: Danzhong (CV17), Zhongwan (CV12), Qihai (CV6), Xuehai (SP10), and Zusanli (ST36) [23]. In previous clinical and experimental studies on VD treated with “Sanjiao” acupuncture, we observed that this acupuncture approach significantly improved both cognitive function and self-care abilities in VD patients [24], and also enhanced learning and memory performance in VD rats [25]. Studies have revealed that the improvement of learning and memory abilities by “Sanjiao” acupuncture is primarily associated with enhanced cerebral blood flow, amelioration of mitochondrial dysfunction [25], promotion of cerebral glucose aerobic metabolism [23], antioxidant effects [25], anti-apoptotic mechanisms [26], anti-inflammatory actions [27], as well as improvement of the brain microenvironment in dementia model rats [28]. Meanwhile, in previous studies, we demonstrated that “Sanjiao” acupuncture improved cognitive function in VD rats and modulated the balance of Th1/Th2 and Th17/Treg cells in peripheral blood [27, 29]. However, the effects of acupuncture on microglial activation and neuroinflammatory levels in the brain, neuronal apoptosis, as well as changes in peripheral T-cell subsets (including proportion, proliferation, and apoptosis) remain poorly understood. Therefore, this study aims to further investigate these aspects.

## Materials and methods

### Animals

Forty healthy male Wistar rats (SPF grade, 250–280 g) [certificate No. SCXK (jing) 2019-0008] were purchased from Beijing HFK Bioscience Co., Ltd. (Beijing, China). The rats were housed under controlled temperature (23 ± 2 °C), humidity (40–70%), and a 12 h light/dark cycle, with free access to food and water. The experiments were approved by the Ethics Committee of the Medical Laboratory Animal Center of Tianjin University of Chinese Medicine (permission number: TCM-LAEC2023217w3389). All procedures complied with the Regulations of Experimental Animal Administration issued by the Ministry of Science and Technology of the People’s Republic of China. Every effort was made to minimize both the number of animals used and their distress.

### Permanent bilateral common carotid artery occlusion

After one week of acclimation, all animals were randomly divided into a sham-operated group (Gs, n = 10) and an operated group (Go, n = 30). The rat model of VD was established by permanent bilateral common carotid artery occlusion (2-VO) [29]. Rats were first rapidly anesthetized with 3% isoflurane mixed with 97% oxygen, and then anesthesia was maintained with 2% isoflurane mixed with 98% oxygen. Through a midline neck incision, the bilateral common carotid arteries were exposed, the vagus nerves were gently separated, and both vessels were ligated with 4–0 silk suture. After ligation, the skin incision was sutured, disinfected, and sprayed locally with 0.2 mL of gentamicin sulfate injection to prevent wound infection. The same surgical procedure, excluding artery ligation, was performed on the Gs group. Throughout the surgery, procedures were conducted as gently as possible to minimize animal pain, and a heating blanket was used to maintain the rats' body temperature.

### Model screening and grouping

Two months after surgery, 20 VD rats were selected based on Morris Water Maze (MWM) results. The screening criteria were as follows: the mean escape latency of the Gs group was used as the reference value. For each rat in the surgery group, the ratio was calculated as (the mean escape latency of the surgically treated rat minus the reference value) divided by the mean escape latency of that same surgically treated rat. Rats with this ratio greater than 20% were defined as dementia models [30]. Subsequently, these VD rats were randomly divided into a model group (Gm, n = 10) and an acupuncture group (Ga, n = 10).

### Acupuncture treatment

**Fig. 1:**
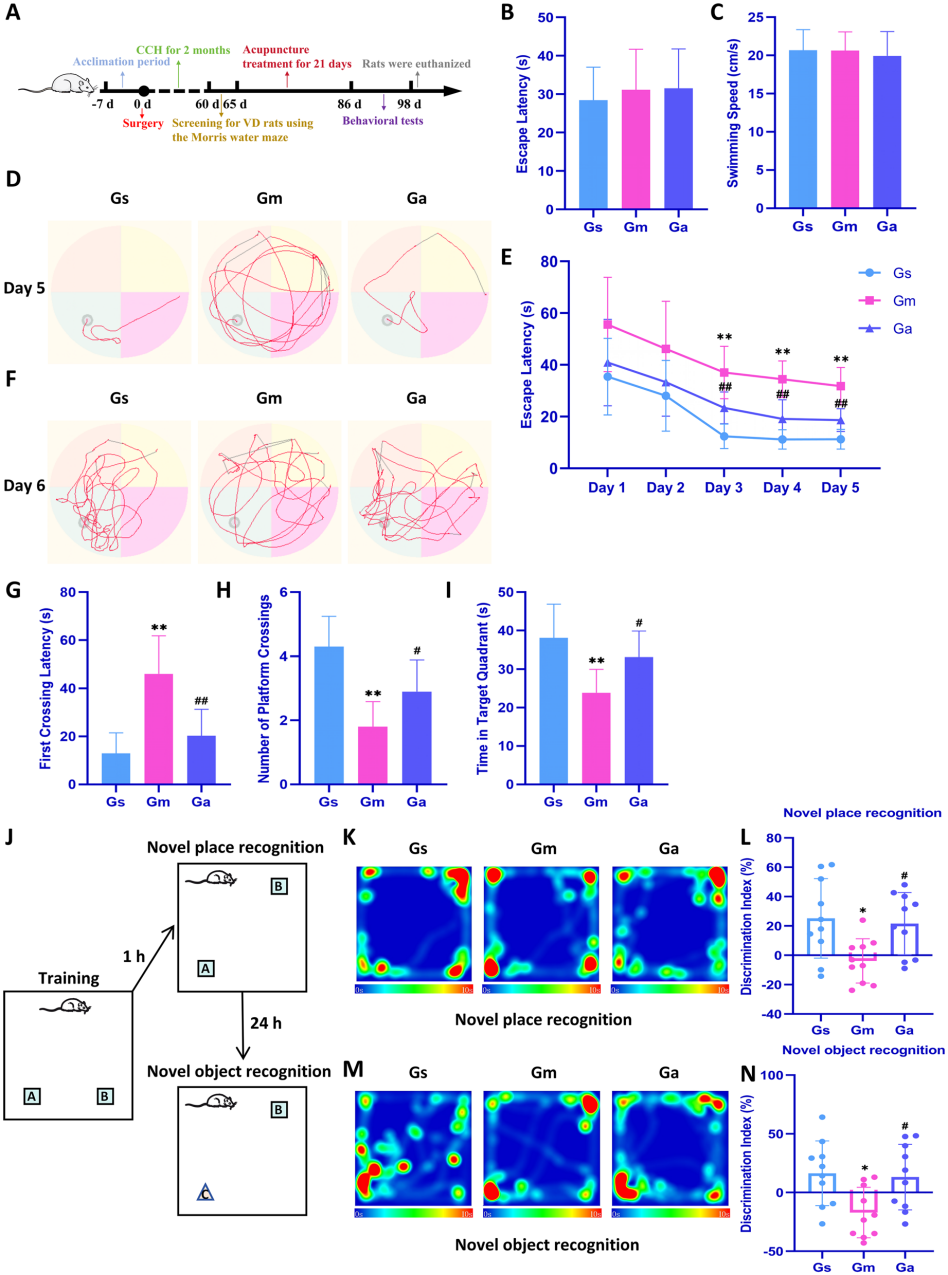
Effects of acupuncture on spatial learning and memory in VD rats. **A** The complete flow chart of this study. **B**, **C** Statistical analysis of escape latency and swimming speed of each group in the visible platform test. **D** Representative trajectories of each group in the hidden platform test on day 5. **E** Escape latency during spatial acquisition training (days 1–5). **F** Representative trajectories of each group in the probe test on day 6. **G**–**I** Latency of first arrival at platform area, number of crossing through platform area, and total time in target quadrant during probe test on day 6 for each group. **J** Schema of novel place location and novel object recognition assays. **K**, **L** Representative heatmaps and discrimination index statistics for each group in the NPR test. **M**, **N** Representative heatmaps and discrimination index statistics for each group in the NOR test. All values are expressed as the mean ± SD (n = 10). ^*^P < 0.05, ^**^P < 0.01 vs. the Gs group; ^#^*P* < 0.05, ^##^*P* < 0.01 vs. the Gm group

Rats in the Ga group underwent “Sanjiao” acupuncture treatment based on a protocol from previous studies [25, 27]. The anatomical localization of rat acupoints was determined with reference to established literature [31, 32]. The specific acupoint locations, depths of insertion, and treatment durations are detailed in Table 1. Disposable sterile acupuncture needles (0.25 × 13 mm, Hwato, China) were used during the experiment. After disinfecting the skin with iodine, needles were inserted horizontally 2–3 mm into CV17; 3–4 mm perpendicularly into CV12, CV6, and ST36; 2–3 mm obliquely into SP10. CV17, CV12, CV6, and bilateral ST36 were needled with a reinforcing twirling technique; bilateral SP10 received a reducing twirling technique [28]. Acupuncture was administered once daily for 21 consecutive days, with one day of rest per week. Rats in the Gs and Gm groups were handled with identical duration and restraint pressure as those in the Ga group, but without acupuncture. The experimental design and schedule are shown in Fig. 1A.

**Table 1:** Name, location, depth, and duration

Points	Location	Depth (mm)	Duration (s)
Danzhong (CV17)	Midline of the abdomen at the level of the fourth intercostal space	2–3	30
Zhongwan (CV12)	Approximately 20 mm above the umbilicus	3–4	30
Qihai (CV6)	In the hypogastric region, 4 mm inferior to the umbilicus along the anterior median line	3–4	30
Xuehai (SP10)	On the medial aspect of the thigh, approximately 4 mm proximal to the superomedial border of the patella	2–3	30
Zusanli (ST36)	On the lateral side posterior to the knee joint, approximately 3 mm inferior to the caput fibula	3–4	30

### MWM test

Spatial learning and memory were evaluated in rats using the MWM. All rats underwent acclimation training the day before experimental testing commenced. Subsequently, a visible platform test was conducted to exclude interference from visual impairment, swimming ability deficits, and differences in physical stamina. Hidden platform test: following the visible platform test, the platform was submerged approximately 2 cm below the water surface. Rats underwent training for 5 days (twice daily), with randomized and non-repeating entry points for each trial. Each rat was allowed a maximum of 90 s to locate the hidden platform, and escape latency was recorded. If a rat failed to find the platform within 90 s, it was guided to the platform by the experimenter and allowed to remain there for 10 s to acclimate to the location; in such cases, the escape latency was recorded as 90 s. Probe test: following the hidden platform test, the platform was removed and rats were allowed to swim freely in the pool for 90 s, during which the following parameters were recorded: the latency to first crossing of the former platform location, time spent in the target quadrant, and number of crossings over the platform location. Reversal test: the platform was relocated to the opposite quadrant, and rats underwent testing for 3 days (twice daily) using the identical protocol as in the hidden platform test.

### Novel place recognition (NPR) and novel object recognition (NOR) test

This process comprised three stages: adaptation, training, and probe trials. On day 1, all rats were individually placed in a 100 × 100 × 40 cm test chamber for 10 min to adapt to the test environment. On day 2, spatial orientation ability was assessed: rats were placed in the test chamber with two identical objects (matched in shape, size, and material) and allowed free exploration for 10 min (training phase). One hour post-training, one object was relocated to a novel place and exploration was conducted for 5 min (test phase). After 24 h (day 3), with the object placements maintained as in day 2's test phase, one object was replaced by a novel object of identical texture but distinct shape placed in its stead; rats then explored freely for 5 min (test phase). Between trials, the arena and objects were cleansed with 70% ethanol to eliminate odor cues. The entire process will be recorded on video equipment. The discrimination index was calculated using the following formula: $\text{discrimination index} = \frac{\text{novel exploration time} - \text{familiar exploration time}}{\text{total exploration time}} \times 100\%$ [33].

### Hematoxylin and eosin (HE) staining and TUNEL staining

Following behavioral testing, rats were anesthetized via intraperitoneal injection of pentobarbital sodium (40 mg/kg) and transcardially perfused with pre-chilled 0.9% saline followed by 4% paraformaldehyde (PFA). Whole brains were removed, post-fixed in 4% PFA for 24 h, then cryoprotected through graded sucrose solutions (10%, 20%, and 30%). Tissues were embedded in OCT compound, flash-frozen in liquid nitrogen, and sectioned coronally at 8 μm thickness using a cryostat. Hippocampal neuronal injury was evaluated by HE staining (Solarbio, Beijing, China). Images were acquired using a Digital Pathology Slide Scanner (KF-FL-005, Konfoong Bioinformation Tech Co., Ltd, China).

TUNEL staining was performed to assess apoptosis levels in hippocampal tissue. Brain sections prepared as described above were processed using a TUNEL staining kit (Beyotime, Shanghai, China) according to the manufacturer's protocol. Images were acquired with a fluorescence microscope (Leica Microsystems, Germany) and quantitatively analyzed using ImageJ software. The neuronal apoptosis rate was calculated using the following formula: $\frac{\text{number of TUNEL-positive cells}}{\text{total number of cells}} \times 100\%$ [34].

### Immunofluorescence staining

Prepare brain sections for immunofluorescence staining as described above. Frozen Sects. (8 μm) were fixed with 4% PFA at room temperature for 30 min, followed by three 5 min washes in PBS (pH 7.4). Sections were incubated with 0.5% Triton X-100 (P1081, Solarbio) for 30 min, blocked with 0.5% BSA (A8020, Solarbio) for 1 h, and then incubated overnight at 4 °C in the dark with the following primary antibodies: mouse anti-Iba-1 (1:500; sc-32725, Santa Cruz) and rabbit anti-CD68 (1:1000; ab283654, Abcam). After washing with PBS, sections were incubated for 2 h at room temperature in the dark with secondary antibodies: CoraLite 594-conjugated donkey anti-mouse IgG (H + L) (1:200; SA00013-7, Proteintech) and Alexa Fluor^®^ 488-conjugated goat anti-rabbit IgG (H&L) (1:1000; ab150077, Abcam). Following another PBS wash, nuclei were counterstained with DAPI for 5 min in the dark. Finally, sections were washed with PBS and coverslipped. Images were acquired using a Digital Pathology Slide Scanner (KF-FL-005, Konfoong Bioinformation Tech Co., Ltd, China) and analyzed with ImageJ (Fiji). Colocalization analysis of CD68 and Iba-1 was performed using the Colocalization Finder plugin in ImageJ (Fiji).

### Western blotting

To quantify apoptosis-related protein expression in rat hippocampal tissue, Western blot analysis was performed. Rats were euthanized via intraperitoneal injection of pentobarbital sodium (2%, 40 mg/kg). Hippocampal tissues were rapidly dissected from rat brains, flash-frozen in liquid nitrogen, and stored at − 80 °C for subsequent analysis. Western blotting was performed with reference to established literature (Wang et al., 2023). Hippocampal tissues were lysed in RIPA buffer (R0010, Solarbio) containing protease inhibitors (PMSF) and phosphatase inhibitors. Protein concentrations were quantified using a BCA assay kit (PC0020, Solarbio), with 30 μg of total protein loaded per sample. Proteins were then resolved by 10% SDS-PAGE and transferred to PVDF membranes. The membrane was blocked with 5% skim milk in TBST for 2 h. After washing with TBST, it was incubated with primary antibody at 4 °C overnight. The following day, the membrane was washed again with TBST and incubated with secondary antibody (1:5000; BS13278, Bioworld Technology) at room temperature for 2 h. Band images were acquired with a ChemiScope 6100 Chemiluminescence Imaging System (Clinx Science Instruments, China), followed by quantitative analysis using ImageJ software (National Institutes of Health, USA). Primary antibodies included anti-cleaved caspase-3 (1:1000; #9661, Cell signaling technology), anti-caspase-3 (1:1000; ab179517, Abcam), anti-Bcl-2 (1:1000; ab196495, Abcam), anti-Bax (1:1000; ab32503, Abcam), and anti-β-Actin (1:5000; AP0060, Bioworld Technology) as an internal control.

### Enzyme-linked immunosorbent assay (ELISA)

After the behavioral tests, hippocampal tissue and serum were collected from the rats. According to the manufacturer’s instructions, inflammatory cytokine concentrations in both hippocampal tissue and serum were measured with ELISA. The cytokines assessed included IL-1β, IL-2, IL-4, IL-6, IL-10, IL-17A, and TNF-α (all kits from Elabscience Biotechnology Co., Ltd., Wuhan, China). TGF-β levels were measured using a kit from Nanjing Jiancheng Bioengineering Institute (Nanjing, China), and IL-35 levels were measured using a kit from Nanjing SenBeiJia Biological Technology Co., Ltd. (Nanjing, China).

### Flow cytometry

The proportions and proliferation of T-cell subsets were analyzed by flow cytometry using venous blood collected from rat tails (the detailed gating strategy is provided in Supplementary Fig. S1). Briefly, venous blood was collected from rat tails into heparinized tubes and lysed with Red Blood Cell Lysis Buffer (R1010, Solarbio) for 10 min to prepare single-cell suspensions. After two washes with 1 × PBS (4 ℃, 450 × g, 5 min), cells were resuspended in Staining Buffer (554656, BD Biosciences) at a final concentration of 1 × 10⁶ cells/mL. Flow cytometry tubes were allocated into four groups: negative controls, single-stain controls, isotype controls, and experimental samples, each containing 100 µL of single-cell suspension. Surface and intracellular stainings were performed following standardized protocols. Cells were surface-stained with APC-Cy7-CD45 (561586, BD Biosciences), PerCP-eFluor710-CD3 (46-0030-82, Invitrogen), FITC-CD4 (11–0040–82, Invitrogen), and PE-Cyanine7-CD8a (25–0084-82, Invitrogen) at 4 °C for 30 min in the dark. After two washes with cold staining buffer (4 ℃, 450 × g, 5 min), cells were fixed and permeabilized using Transcription Factor Buffer (562574, BD Biosciences), followed by intracellular staining with Ki67 antibody (69–5698-82, Invitrogen) at 4 °C for 40 min in the dark. Finally, cells were washed twice in staining buffer (4 ℃, 500 × g, 6 min) and analyzed on a BD FACSymphony™ A1 Cell Analyzer, acquiring at least 10,000 events. Gating strategies employed single-stain controls and isotype controls, with data analyzed using FlowJo 10.6.2 software.

T-cell subset apoptosis was analyzed by flow cytometry using the same protocol described above, with gating strategies detailed in Supplementary Fig. S2. Following surface staining with APC-Cy7-CD45 (561586, BD Biosciences), FITC-CD3 (559975, BD Biosciences), R718-CD4 (751870, BD Biosciences), and PE-Cyanine7-CD8a (25–0084-82, Invitrogen), cells were washed and stained with PE Annexin V Apoptosis Detection Kit I (559763, BD Biosciences) according to the manufacturer's instructions. Data acquisition was performed by flow cytometry.

### Statistical analysis

Data were analyzed using SPSS 26.0 and GraphPad Prism 8.0. Normality was assessed by the Shapiro–Wilk test, and all data are presented as mean ± standard deviation (SD). For the hidden platform and reversal platform tests, two-way repeated-measures ANOVA was applied; other data were analyzed by one-way ANOVA. Results with *P* < 0.05 were considered statistically significant.

## Results

### Acupuncture treatment improved spatial learning and memory in VD rats

After 21-day acupuncture treatment, cognitive ability in rats was evaluated using MWM, NPR, and NOR tests.

In the MWM test, a visible platform test was first conducted for all rats. No significant differences in escape latency or swimming speed were observed between groups (Fig. 1B, C), indicating no interference from vision, swimming ability, or physical fitness. Then, a hidden platform test was performed on all rats. As shown in Fig. 1D, after 5 days of training, rats in the Gm group still exhibited random search patterns, whereas those in the Gs and Ga groups adopted more targeted strategies. Analysis of escape latency during the hidden platform phase (Fig. 1E) revealed that as training progressed, latency significantly decreased in Gs and Ga groups, showing statistically significant differences compared to the Gm group from day 3 onward. The probe test was conducted on day 6, with results presented in Fig. 1F–I. Compared to the Gs group, rats in the Gm group exhibited significantly prolonged first platform-crossing latency, reduced dwell time in the target quadrant, and fewer platform crossings. Acupuncture treatment significantly improved these spatial memory impairments. Finally, a reverse platform test was conducted to evaluate the rats' relearning and spatial memory abilities. Compared to the Gs group, the Gm group showed prolonged escape latency. However, post-acupuncture treatment significantly shortened latency (Supplementary Fig. S3).

Following the MWM test, NPR and NOR tests were conducted. In the NPR test, compared to the Gs group, the Gm group showed significantly reduced novel place exploration time, which was significantly improved by acupuncture treatment (Fig. 1K, L). In the NOR test, Gm rats exhibited a significantly lower discrimination index compared to Gs rats, whereas the acupuncture-treated group demonstrated enhanced novelty discrimination (Fig. 1M, N).

The above data indicate that acupuncture treatment significantly improves the spatial learning and memory abilities in VD rats.

### Acupuncture treatment reduces neuronal injury and apoptosis in the hippocampus of VD rats

**Fig. 2:**
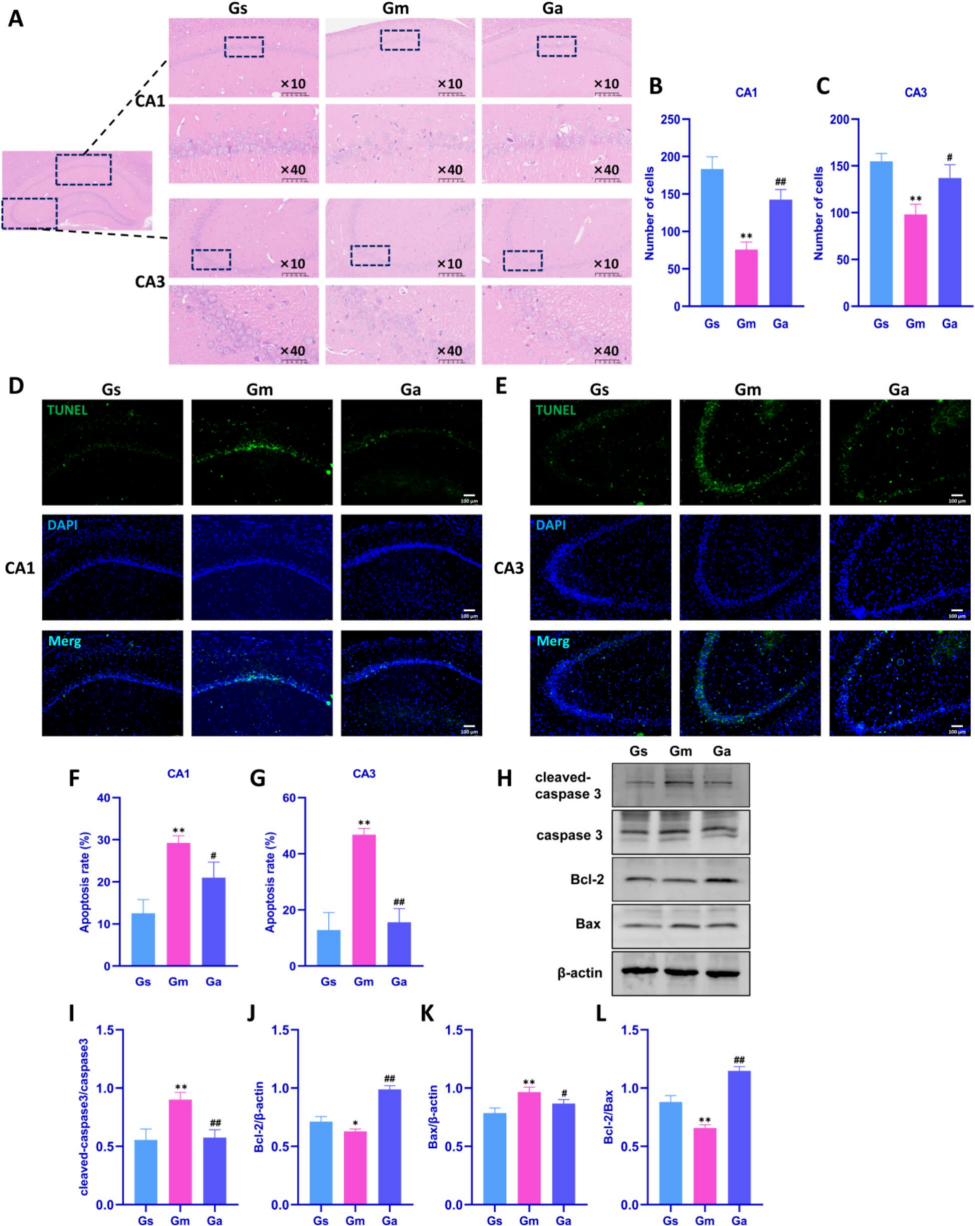
Effects of acupuncture on hippocampal neuronal injury and apoptosis in VD rats. **A** Morphology of hippocampal CA1 and CA3 neurons detected by HE staining in each group. Imaged at 10 × magnification, scale bar = 200 μm; representative region at 40 ×, scale bar = 50 μm. **B**, **C** Results of neuronal cell count in hippocampal CA1 and CA3 regions measured by HE staining (n = 3). **D**-**G** Representative images and quantitative analysis of TUNEL-positive cells (green) and DAPI-stained nuclei (blue) in hippocampal CA1 and CA3 regions. Scale bar: 100 μm; n = 3. **H**-**L** Representative western blot bands and analysis of cleaved-caspase 3, caspase 3, Bcl-2 and Bax in hippocampus of each group (n = 4). β-actin was used as loading control. All values are expressed as the mean ± SD. ^*^P < 0.05, ^**^P < 0.01 vs. the Gs group; ^#^*P* < 0.05, ^##^*P* < 0.01 vs. the Gm group

Numerous studies indicate that cognitive impairment is closely associated with hippocampal neuronal apoptosis [12]. To determine acupuncture's effects on hippocampal neurons in VD rats, we first observed neuronal morphology in CA1 and CA3 regions via HE staining. As shown in Fig. 2A–C, the Gm group exhibited cytoplasmic loss and irregular nuclear aggregation. Acupuncture treatment significantly ameliorated neuronal damage in CA1 and CA3 regions.

Subsequently, TUNEL staining was performed to detect apoptosis in the hippocampus (CA1 and CA3 regions) of VD rats, and TUNEL-positive cells were quantified. As shown in Fig. 2D–G, compared to the Gs group, the Gm group exhibited significantly increased neuronal apoptosis in CA1 and CA3. Following acupuncture treatment, the Ga group showed significantly reduced apoptosis in these regions compared to the Gm group. Meanwhile, Western blotting was performed to detect cleaved-caspase 3, caspase 3, Bax, and Bcl-2 expression in rat hippocampal tissues. As shown in Fig. 2H–L, compared with the Gs group, the Gm group showed significantly increased expression of pro-apoptotic factors cleaved-caspase 3 and Bax in the hippocampus, significantly decreased expression of anti-apoptotic factor Bcl-2, and a reduced Bcl-2/Bax ratio. Acupuncture treatment significantly inhibited pro-apoptotic factor expression (cleaved-caspase 3, Bax), promoted anti-apoptotic Bcl-2 expression, and increased the Bcl-2/Bax ratio. These results indicate that acupuncture ameliorates CCH-induced neuronal apoptosis in the hippocampus of VD rats.

### Acupuncture treatment inhibits hippocampal neuroinflammation in VD rats

**Fig. 3:**
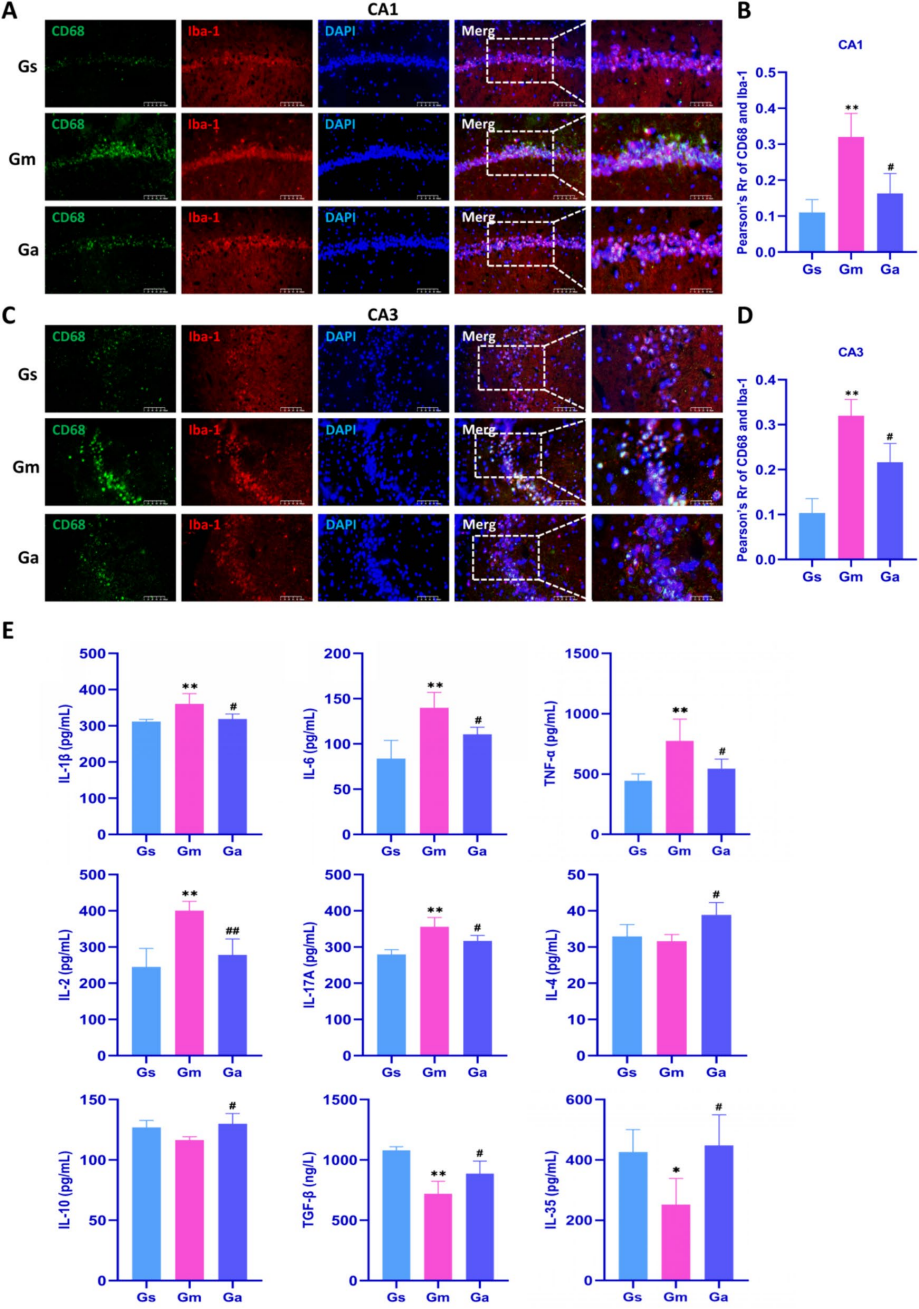
Effects of acupuncture on microglial markers Iba-1 and CD68 in the hippocampus of VD rats. **A** Representative images of immunofluorescence for Iba-1 (red), CD68 (green), and DAPI (blue) in the hippocampal CA1 region of each group. Imaged at 200 × magnification, scale bar = 100 μm; representative region at 400 ×, scale bar = 50 μm. **B** Immunofluorescence colocalization analysis of Iba-1 and CD68 in the hippocampal CA1 region among groups. **C** Representative images of immunofluorescence for Iba-1 (red), CD68 (green), and DAPI (blue) in the hippocampal CA3 region of each group. **D** Immunofluorescence colocalization analysis of Iba-1 and CD68 in the hippocampal CA3 region among groups. **E** Alterations in the levels of inflammatory cytokines (IL-1β, IL-6, TNF-α, IL-2, IL-17A, IL-4, IL-10, TGF-β, and IL-35) in the hippocampal tissue among each group of rats. All values are expressed as the mean ± SD (n = 4). ^*^P < 0.05, ^**^P < 0.01 vs. the Gs group; ^#^*P* < 0.05, ^##^*P* < 0.01 vs. the Gm group

As resident immune cells in the brain, microglia mediate neuroinflammation, which plays an important role in VD-related brain injury [11]. To clarify acupuncture's effects on neuroimmune responses in VD rats, we performed immunofluorescence staining to examine colocalization of microglial markers Iba-1 and CD68 in hippocampal CA1 and CA3 regions. As shown in Fig. 3A–D, compared with the Gs group, the numbers of Iba-1^+^ and CD68^+^ cells in the CA1 and CA3 regions of the hippocampus were significantly increased in the Gm group. Acupuncture treatment significantly down-regulated Iba-1 and CD68 expression in these regions. Using ImageJ (Fiji) software, fluorescence colocalization analysis between Iba-1 and CD68 was performed, and Pearson's correlation coefficient (R) was calculated. Significantly reduced Iba-1/CD68 colocalization in microglial cells was observed in the hippocampal CA1 and CA3 regions of Ga rats versus Gm rats (Fig. 3B, D).

To further explore the effects of acupuncture on neuroinflammation, we used ELISA kits to assess cytokine levels (IL-1β, IL-6, TNF-α, IL-2, IL-17A, IL-4, IL-10, TGF-β, and IL-35) in the hippocampus. As shown in Fig. 3E, hippocampal concentrations of IL-1β, IL-6, TNF-α, IL-2, and IL-17A were significantly elevated in the Gm group versus the Gs group. Conversely, TGF-β and IL-35 levels were markedly reduced, and IL-4 and IL-10 concentrations were decreased. After acupuncture treatment, the Ga group showed significantly reduced pro-inflammatory cytokine levels (IL-1β, IL-6, TNF-α, IL-2, IL-17A) and elevated anti-inflammatory cytokine levels (IL-4, IL-10, TGF-β, IL-35) compared to the Gm group.

In summary, these findings demonstrate that acupuncture suppresses microglial hyperactivation and ameliorates neuroinflammation in the hippocampus of VD rats.

### Acupuncture treatment regulates inflammatory cytokine levels in the peripheral blood of VD rats

**Fig. 4:**
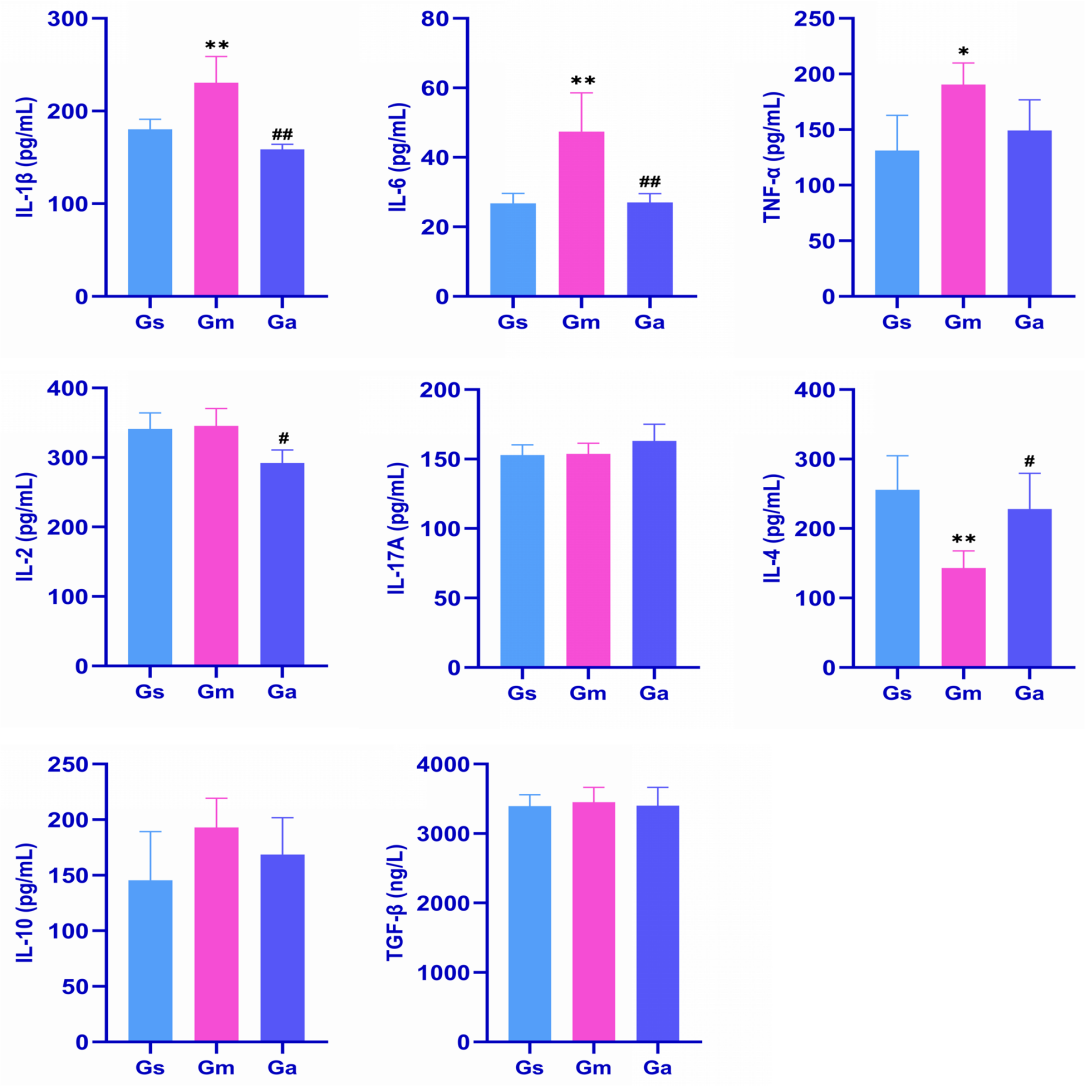
Effects of acupuncture on inflammatory cytokine levels (IL-1β, IL-6, TNF-α, IL-2, IL-17A, IL-4, IL-10, and TGF-β) in peripheral blood of VD rats. All values are expressed as the mean ± SD (n = 4). ^*^P < 0.05, ^**^P < 0.01 vs. the Gs group; ^#^*P* < 0.05, ^##^*P* < 0.01 vs. the Gm group

It is noteworthy that accumulating evidence indicates VD resulting from cerebral ischemia not only triggers central inflammation but also induces peripheral inflammation and broad alterations in the immune system [13, 14]. The levels of IL-1β, IL-6, TNF-α, IL-2, IL-17A, IL-4, IL-10, and TGF-β in the peripheral blood of rats were measured by ELISA. Results: As shown in Fig. 4, the concentrations of IL-1β, IL-6, and TNF-α in the peripheral blood of the Gm group were significantly elevated compared to those in the Gs group. Conversely, the concentration of IL-4 was significantly reduced. Following acupuncture treatment, the Ga group showed significantly reduced levels of IL-1β, IL-6, and IL-2 and elevated IL-4 levels compared to the Gm group.

### Acupuncture treatment regulates the proportions of peripheral T-cell subsets in VD rats

To further investigate peripheral immune changes after CCH and acupuncture's effects, we used flow cytometry to analyze the proportions, proliferation, and apoptosis of T-cell subsets in the peripheral blood of rats.

**Fig. 5:**
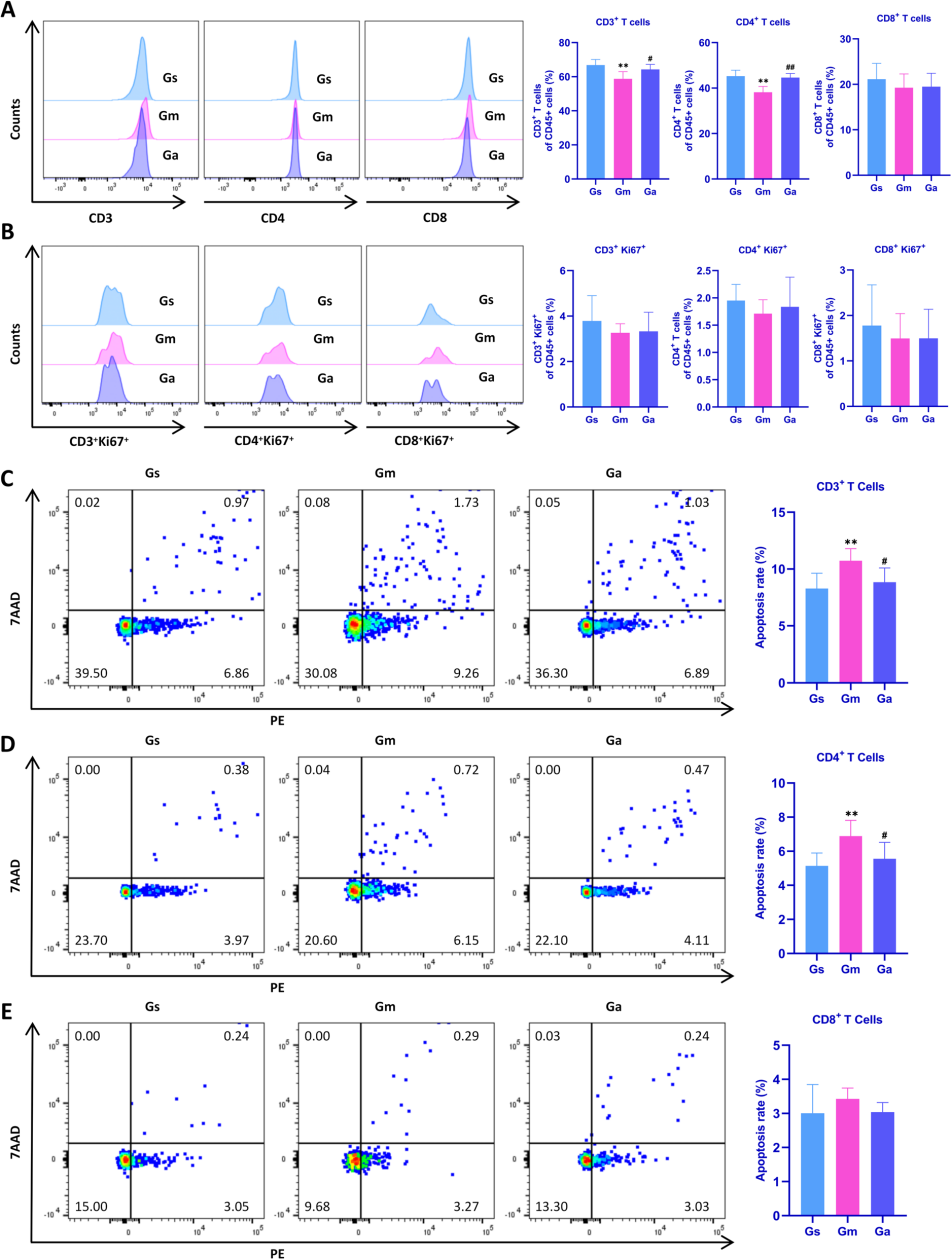
Effects of acupuncture on peripheral blood T-cell subset distribution in VD rats. **A** Percentages of CD3⁺ T cells, CD4⁺ T cells, and CD8⁺ T cells. **B** Percentages of CD3⁺Ki67⁺, CD4⁺Ki67⁺, and CD8⁺Ki67⁺ proliferating cells. **C** Apoptosis of CD3⁺ T cells (percentage of CD45⁺ cells). **D** Apoptosis of CD4⁺ T cells (percentage of CD45⁺ cells). **E** Apoptosis of CD8⁺ T cells (percentage of CD45⁺ cells). All values are expressed as the mean ± SD (n = 6).  ^**^P < 0.01 vs. the Gs group; ^#^*P* < 0.05, ^##^*P* < 0.01 vs. the Gm group

As shown in Fig. 5A, compared with the Gs group, the proportions of CD3^+^ T-cells and CD4^+^ T-cells in the peripheral blood of the Gm group were significantly decreased, while CD8^+^ T-cells showed no significant differences across groups. After acupuncture treatment, the Ga group exhibited significantly increased proportions of CD3^+^ T-cells and CD4^+^ T-cells in peripheral blood. Regarding proliferation, no significant differences were observed in CD3^+^Ki67^+^, CD4^+^Ki67^+^, and CD8^+^Ki67^+^ T-cells in the peripheral blood across groups (Fig. 5B). Regarding apoptosis, compared with the Gs group, apoptosis rates of CD3^+^ T-cells and CD4^+^ T-cells in the peripheral blood of the Gm group were significantly increased, while CD8^+^ T-cell apoptosis showed no significant differences across groups. Acupuncture treatment significantly reduced apoptosis of CD3^+^ and CD4^+^ T-cells in VD rats (Fig. 5C–E).

The results demonstrate that acupuncture up-regulates the proportions of CD3^+^ and CD4^+^ T-cells in the peripheral blood of VD rats, reduces their apoptosis, and ameliorates CCH-induced peripheral immune dysregulation.

## Discussion

This study investigated acupuncture's neuroprotective effects and central-peripheral inflammatory regulation in VD rats. Results demonstrated that acupuncture significantly ameliorated CCH-induced neuronal damage and spatial learning/memory impairments. It not only suppressed hippocampal microglial activation, reduced pro-inflammatory cytokine release, and attenuated neuronal apoptosis, but also decreased peripheral inflammatory cytokines, modulated T-cell subpopulation proportions and apoptosis, and improved peripheral immune function. The acupuncture-mediated cognitive improvement in VD rats involved reduced central inflammation and enhanced peripheral immune regulation.

VD refers to a syndrome of cognitive dysfunction resulting from cerebrovascular diseases (e.g., cerebral ischemia, hemorrhage, or acute/chronic cerebral hypoxia) [35]. Currently, there are no established pharmacotherapies or treatment strategies to effectively prevent and manage VD. As a complementary and alternative therapy, acupuncture is widely accepted among patients, particularly for neurological disorders such as stroke and dementia [36, 37]. In our previous research, we discovered that acupuncture had outstanding clinical efficacy and could significantly ameliorate cognitive impairments in VD patients, including memory, orientation, and computational ability [24]. Moreover, studies have demonstrated that acupuncture enhances cerebral blood flow (CBF), promotes remyelination, and improves cognitive function in VD rats [25, 38]. In the current study, we assessed cognitive function in rats using the MWM, NPR, and NOR tests. The results demonstrated that acupuncture significantly alleviated cognitive impairment in VD rats. Our findings are consistent with existing research [25], further confirming the therapeutic efficacy of acupuncture for vascular dementia.

Neuroinflammation is recognized as a major contributor to brain injury and cognitive dysfunction following CCH. Reduced CBF triggers an ischemic cascade, which activates various brain cells including microglia [11]. As the brain's resident immune cells, microglia orchestrate neuroinflammatory responses. Accumulating evidence indicates that CCH disrupts central nervous system (CNS) homeostasis, prompts microglial accumulation, activation, and polarization at ischemic sites [6, 7]. M1-polarized microglia secrete pro-inflammatory cytokines (IL-1β, TNF-α, IL-6), whereas M2-polarized microglia release anti-inflammatory cytokines (IL-4, IL-10, TGF-β) [8, 9]. Our investigation revealed that in 2-VO-induced VD rats, hippocampal expression of Iba-1 and CD68 was significantly upregulated, accompanied by elevated levels of pro-inflammatory cytokines (IL-1β, IL-6, TNF-α, and IL-17A) and diminished levels of anti-inflammatory cytokines (TGF-β and IL-35). Acupuncture intervention effectively reversed these pathological alterations. The study demonstrates that acupuncture suppresses hippocampal inflammation in VD rats, a mechanism closely associated with the modulation of microglial polarization.

Microscopy revealed that acupuncture significantly attenuated neuronal injury and apoptosis in hippocampal CA1 and CA3 regions. Studies indicate that glial cell-derived inflammatory cytokines (e.g., IL-1β, TNF-α) trigger neuronal apoptosis through modulation of Bcl-2 family proteins and caspases, ultimately driving cognitive decline [39]. As key apoptotic regulators, Bcl-2 family members indirectly control caspase activation. Specifically, the expression balance of Bcl-2 (anti-apoptotic) and Bax (pro-apoptotic) governs apoptotic initiation. The executioner caspase-3 amplifies downstream apoptotic cascades upon activation [40]. Evidence indicates that acupuncture attenuates neuronal damage, apoptosis, and myelin loss in the cerebral cortex and hippocampus [26, 41]. Based on this evidence, we quantified hippocampal neuronal injury and apoptosis in VD rats using HE staining, TUNEL assay, and Western blot analysis. Results demonstrated that acupuncture significantly reduced neuronal apoptosis, concomitant with downregulation of pro-apoptotic effectors (cleaved-caspase 3, Bax) and upregulation of anti-apoptotic Bcl-2. These changes indicate neuroprotective effects against hippocampal neuronal injury in VD.

Recent studies demonstrate that VD patients exhibit not only neuroinflammation but also peripheral inflammation and immune dysregulation [13]. Peripheral inflammation elevates brain pro-inflammatory cytokines (e.g., IL-1β, TNF-α), exacerbating neuroinflammation-associated cognitive deficits in encephalopathy patients [17]. Consistently, elevated peripheral pro-inflammatory cytokines (IL-1β, TNF-α, IL-2) have been reported in dementia patients. Our data confirm peripheral immune dysregulation in VD rats, manifested by increased pro-inflammatory cytokines (IL-1β, IL-6, TNF-α) and decreased anti-inflammatory IL-4. Acupuncture ameliorated this cytokine imbalance.

Meanwhile, we observed that no significant changes were detected in the levels of IL-17A, TGF-β, and IL-10 in the peripheral blood of VD rats, which may represent an intriguing biological phenomenon. We have conducted in-depth analysis and discussion on this observation, and suggest that the following two aspects may account for these results: (1) their intrinsic biological properties. For instance, IL-1β and IL-6 are primarily derived from innate immune cells (such as macrophages and monocytes). As central mediators of innate immunity-dominated systemic inflammation, they rapidly recognize danger signals and are released in large quantities into the bloodstream upon activation of innate immune cells. Moreover, the release of IL-1β and IL-6 involves efficient and direct signaling pathways, supported by potent positive feedback mechanisms (e.g., TNF-α can induce IL-6 production, and IL-1β is also capable of stimulating IL-6 expression), which collectively contribute to their readily detectable changes in concentration [42]. In contrast, IL-17 is primarily produced by local T cells within tissues and acts as an effector cytokine in adaptive immunity-driven local tissue inflammation. Its function is more focused on regulating localized responses, executing “precise strikes” within specific tissue sites without the need for substantial release into the systemic circulation. Furthermore, members of the IL-17 family (such as IL-17A to F) exhibit functional redundancy in the periphery. Inhibition of one member may be compensated by others, resulting in minimal fluctuation in overall levels and thus less pronounced changes in peripheral blood concentration [43]. A study found that peripheral cytokines, including IL-17A, had little predictive value for the recovery of cognitive function during inpatient rehabilitation after stroke [44]. Another study reported no significant changes in serum IL-17 concentrations before and after epileptic seizures [45]. These findings are consistent with the results of the present study in this aspect. (2) Influenced by other factors as well as temporal and spatial specificity, some peripheral inflammatory cytokines may exhibit non-significant changes upon detection. It has been reported that serum IL-10 levels did not change in aged rats after 18 weeks of running [46]. In contrast, another study found a reduction in hippocampal IL-10 in old mice following 8 weeks of running [47]. These seemingly discrepant effects of exercise on IL-10 may need to be considered within the context of the respective microenvironment.

Similarly, in the present study, the effects of acupuncture may also be attributed to the source of the factors, their intrinsic specificity, and their mechanisms of action. Further investigation is warranted in the future; multi-timepoint detection and more in-depth mechanistic studies may help clarify this interesting phenomenon.

Studies demonstrate that cerebral ischemia-induced cognitive decline correlates with altered peripheral T-cell immunity, characterized by reduced CD4⁺ regulatory T (Treg) cells and expanded CD8⁺ effector T cells [13, 48]. Peripheral T-cell deficiency impairs murine cognition, which can be reversed by T-cell transplantation; similarly, T-cell vaccines ameliorate drug-induced cognitive and behavioral deficits [18]. CD4⁺ T cells maintain hippocampal plasticity and neurogenesis—adoptively transferred CD4⁺ T cells exert neuroprotective effects in aging neurons [19]. Previous studies have documented reduced proportions of CD3⁺ T cells and CD4⁺ T cells in VD patients [13]. Extending these findings, we evaluated acupuncture's modulation of peripheral T-cell homeostasis—proportions, proliferation, and apoptosis—in a VD rat model. Acupuncture treatment elevated peripheral CD3⁺ and CD4⁺ T-cell percentages and suppressed apoptosis.

Previous studies have indicated that acupuncture improves cognitive function in VD rats primarily through multiple mechanisms, including reducing levels of inflammatory cytokines in the hippocampus [29], inhibiting neuronal apoptosis in the hippocampal region [26], decreasing reactive oxygen species generation [25], enhancing cerebral blood flow [25], as well as promoting angiogenesis and myelin repair [41]. These findings collectively demonstrate the regulatory effects of acupuncture on the CNS in VD rats. However, few studies have focused on peripheral alterations in VD or the modulatory role of acupuncture on peripheral immune responses. In this study, we found that acupuncture can modulate the proportion and apoptosis of peripheral CD4⁺ T cells, improve peripheral immune function, and alleviate neuroinflammation, thereby enhancing cognitive performance. Our research explores the mechanisms of acupuncture in treating VD from a central-peripheral interaction perspective, offering new insights into the functional mechanisms of acupuncture and its clinical application for VD. However, the specific underlying mechanisms still require further investigation.

Research has demonstrated that rat models of cerebral ischemia display both BBB disruption and infiltration of peripheral immune cells into the brain [49, 50]. Similarly, in our previous study, we have observed increased activity of Th17 cells and decreased activity of Treg cells in the brains of VD rats. Acupuncture was found to modulate the Th17/Treg balance, thereby improving cognitive function [27]. Additionally, studies have reported a decline in both the quantity and function of Treg cells in the peripheral blood of patients with VD [48]. Treg cells play a key role in suppressing inflammatory responses and maintaining immune homeostasis [51]. Studies have shown that Treg cells exert regulatory effects on CD4^+^ T cells and their subsets. Under pathological conditions, Treg cells can inhibit the functions of Th1, Th2, and Th17 cells, thereby modulating neuroinflammation [52]. Intravenous infusion of in vitro-expanded Treg cells has been shown to ameliorate neuroinflammatory responses in rat models of cerebral ischemia [53]. Therefore, taken together with existing evidence, Treg cells may play a crucial role in modulating inflammatory responses and alleviating neurological damage in VD. Further investigation into Treg cells and their associated mechanisms will remain a primary focus of our ongoing research, which may provide new insights into the interplay between the CNS and peripheral immunity.

## Conclusions

Acupuncture significantly ameliorated CCH-induced cognitive deficits, inhibited neuroinflammation, and reduced neuronal apoptosis. These neuroprotective effects may be mediated, at least partially, through acupuncture-induced attenuation of peripheral inflammation, modulation of T-cell subpopulations, and suppression of apoptosis.

## Supplementary Information

Supplementary Material 1

## Data Availability

The datasets used and/or analyzed during the current study are available from the corresponding authors on reasonable request.

## Abbreviations

VD: Vascular dementia
2-VO: Permanent bilateral common carotid artery occlusion
BBB: Blood–brain barrier
CCH: Chronic cerebral hypoperfusion
LPS: Lipopolysaccharide
IL-1β: Interleukin-1β
TNF-α: Tumor necrosis factor-alpha
MWM: Morris water maze
PFA: Paraformaldehyde
NPR: Novel place recognition
NOR: Novel object recognition
HE: Hematoxylin and eosin
ELISA: Enzyme-linked immunosorbent assay
CBF: Cerebral blood flow
