# Supplementary material for: Acupuncture reduces neuroinflammation and apoptosis, regulates peripheral immunity, and modulates T-cell subset distribution in vascular dementia rats
Source: Chin Med. 2025 Nov 26;20:203. doi: 10.1186/s13020-025-01243-6 (PMC12648782; doi:10.1186/s13020-025-01243-6)
Supplement: Supplementary file 1 — Supplementary Material 1 [file 13020_2025_1243_MOESM1_ESM.docx]

**Supplementary Material**

**
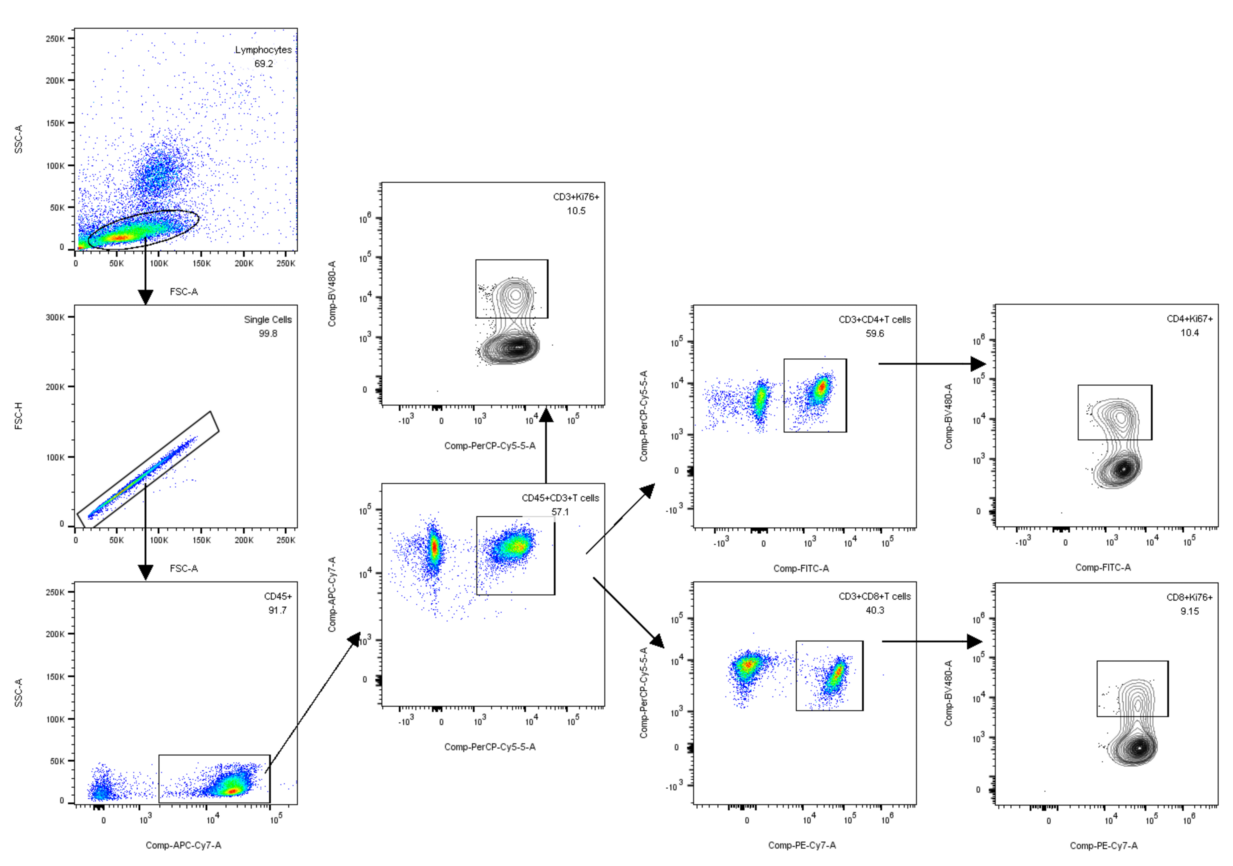
**

**Fig. S1** Gating strategy for identifying T cell subsets and assessing proliferation.

**
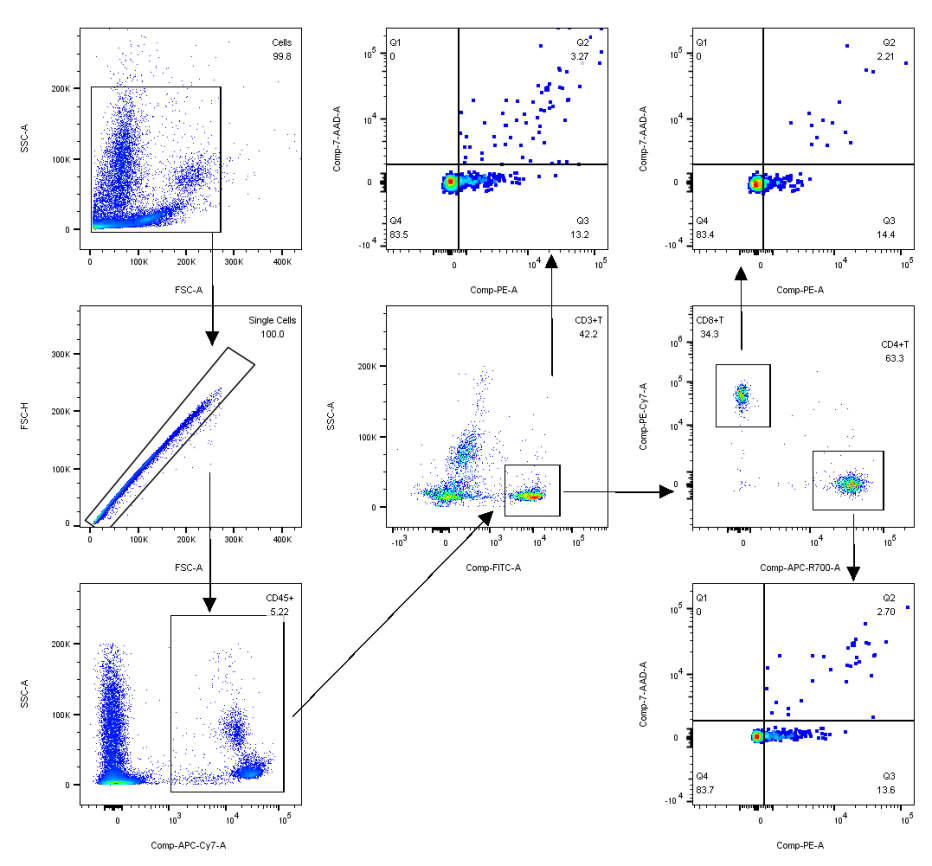
**

**Fig. S2** Gating strategy for the quantification of apoptosis in T cell subsets.

**
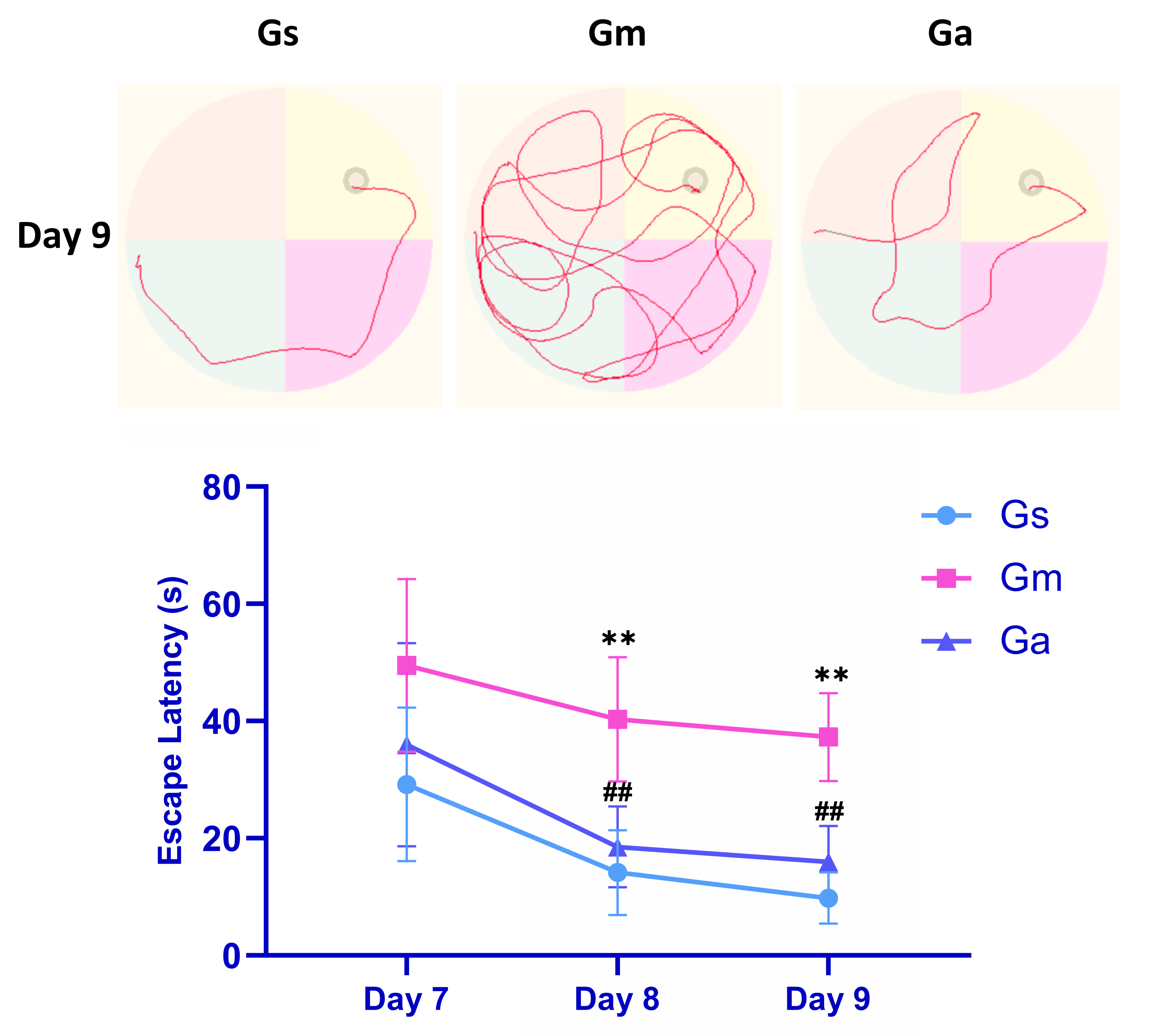
**

**Fig. S3** Representative trajectories of each group in the hidden platform test on day 9 and escape latency during reversal training (days 7–9).
